# The Structure and Occurrence of a Velum in *Utricularia* Traps (*Lentibulariaceae*)

**DOI:** 10.3389/fpls.2019.00302

**Published:** 2019-03-22

**Authors:** Bartosz J. Płachno, Piotr Świątek, Vitor F. O. Miranda, Piotr Stolarczyk

**Affiliations:** ^1^ Department of Plant Cytology and Embryology, Institute of Botany, Jagiellonian University in Kraków, Cracow, Poland; ^2^ Department of Animal Histology and Embryology, University of Silesia in Katowice, Katowice, Poland; ^3^ Faculdade de Ciências Agrárias e Veterinárias, Jaboticabal, Departamento de Biologia Aplicada à Agropecuária, UNESP–Universidade Estadual Paulista, São Paulo, Brazil; ^4^ Unit of Botany and Plant Physiology, Institute of Plant Biology and Biotechnology, University of Agriculture in Kraków, Cracow, Poland

**Keywords:** carnivorous plants, *Utricularia*, trap, functional morphology, functional anatomy, *Bivalvaria*, *Polypompholyx*

## Abstract

Bladderworts (*Utricularia*, *Lentibulariaceae*, Lamiales) are carnivorous plants that form small suction traps (bladders) for catching invertebrates. The velum is a cuticle structure that is produced by specialized trichomes of the threshold pavement epithelium. It is believed that the velum together with the mucilage seals the free edge of the trap door and that it is necessary for correct functioning of the trap. However, recently, some authors have questioned the occurrence of a velum in the traps of the *Utricularia* from the various sections. The main aim of this study was to confirm whether velum occurs in the traps of the *Utricularia* species from the subgenera *Polypompholyx*, *Bivalvaria*, and *Utricularia*. The 15 species were examined from subg. *Polypompholyx*, subg. *Bivalvaria*, and subg. *Utricularia*. A velum was found in all examined *Utricularia* species. In the traps of the members of section *Pleiochasia*, there was an outer velum (forming a complete ring) and an inner velum. In the traps of *Utricularia uniflora* (*Lasiocaules*), there was only an inner velum. In these species, the formation of the velum was accompanied by intensive mucilage production, and as a result, when door was closed (set position), the mucilage and the velum touched the surface of the door. In members of both sections of *Pleiochasia* and *Lasiocaules*, the pavement epithelium had a more complicated structure (four to five zones) than in the members of the subgenera *Bivalvaria* and *Utricularia* in which three distinct zones occurred (an outer with a velum, a middle and an internal with the mucilage trichomes). Even in *U. purpurea*, where the threshold was a reduced pavement epithelium, it consisted of three functional zones and the presence of a velum. Two main types of velum have been proposed. A velum was present in *Utricularia* traps regardless of the trap type or the habitat (aquatic, epiphytic, and terrestrial species). We proposed broad definition of velum as cuticle membranes covered by mucilage; from a functional point of view, this definition is more useful and more reflects complexity of this structure.

## Introduction

**Figure 1:**
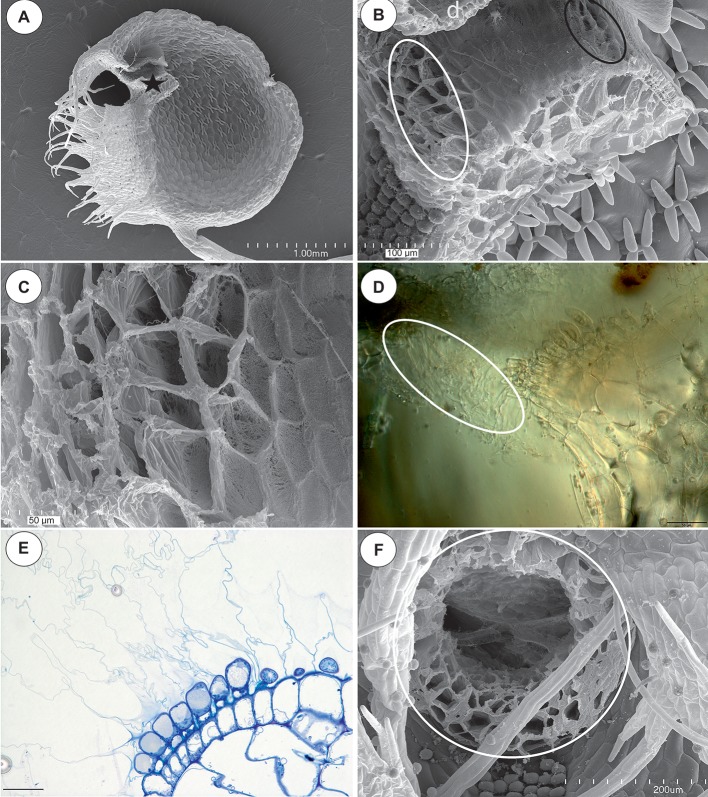
The general structure of the trap and velum of *Utricularia novae-zelandiae*. **(A)** SEM image of a longitudinal section of an *Utricularia novae-zelandiae* trap: the threshold with the pavement epithelium (star), door (d); scale bar = 1 mm. **(B)** A longitudinal section of a *Utricularia novae-zelandiae* trap entrance, note the outer velum (white ellipse, this part is zoomed in Figure 2C) and inner velum (black ellipse); scale bar = 100 μm. **(C)** The outer velum, note the honeycomb-like structure of the velum; scale bar = 50 μm. **(D)** Differential interference contrast microscopy; *Utricularia novae-zelandiae* trap entrance, note the outer velum (white eclipse); scale bar = 50 μm. **(E)** A semi-thin longitudinal section of the threshold of the external part showing the trichomes that produce the external velum; scale bar = 20 μm. **(F)** View of a trap entrance showing the ring of the velum (white circle); scale bar = 200 μm.

Bladderworts (*Utricularia* spp., *Lentibulariaceae*, Lamiales) have suction traps (“bladders”) for catching invertebrates (i.e., Mette et al., 2000; Alkhalaf et al., 2009; Płachno et al., 2014, 2015; Darnowski et al., 2018; Płachno and Muravnik, 2018), and sometimes, also for bacteria and protozoa cultures (Sirová et al., 2009, 2018; Płachno et al., 2012). They are considered to be the fastest predators in the plant kingdom (Vincent and Marmottant, 2011; Adamec, 2011a,b, 2012; Vincent et al., 2011a,b; Poppinga et al., 2013, 2016, 2017). The *Utricularia* trap is a small hollow bladder with a trap entrance (Figure 1A). The lower part of the trap entrance is termed the threshold. This massive, collar-like structure has an agglomeration of tightly packed glandular trichomes on the surface of its middle region (Figures 1A,B). This agglomeration of trichomes is called the pavement epithelium (for a detailed description of the threshold structure see Lloyd, 1942; Poppinga et al., 2016).

Lloyd (1942) recognized three zones in the pavement epithelium: outer, middle, and inner in the traps of *Utricularia vulgaris*. The trichomes from all of the zones have ruptured cuticles. However, the cuticles of the trichomes of the outer zone are very well developed and form balloon-like structures, which together form the velum. Moreover, the cuticles of the trichomes from the middle zone are attached to the cuticles of the trichomes from the outer zone and participate in forming the velum. According to this author, the velum forms a valve that seals the free edge of the trap door. Lloyd (1932a) did an experiment and cut the velum. The traps without a velum could not reset. Thus, the occurrence of a velum is necessary for correct functioning of a trap.

It should be stressed that Lloyd (1942) observed a velum in the traps of various *Utricularia* species (also in members of *Polypompholyx*), regardless of the trap types or the habitat: aquatic, epiphytic, and terrestrial species. According to him, in the traps of *Utricularia monanthos* and related species (section *Pleiochasia*), there is an outer and inner velum. The velum in this type of trap forms a complete massive ring (see Lloyd, 1942).

The structure of the pavement epithelium and the velum was later analyzed by Broussaud and Vintejoux (1982) (*U. vulgaris* L., *Utricularia australis* R.Br.), Fineran (1985: *U. monanthos* Hook.f., which is treated by some authors as a synonym of *Utricularia dichotoma* Labill.), and Heide-Jørgensen (1989, 1991: *Utricularia sandersonii* Oliv., *U. australis*, *Utricularia reniformis* A.St-Hil., *U. subulata* L.), who used transmission electron microscopy. Fineran (1985) proposed that a well-developed velum is a character of the *U. vulgaris* type of trap and that this might represent an advanced structural feature that is connected with the position of door against the threshold. According to him, the concept of a velum in traps other than those of the *U. vulgaris* type should be studied in more detail. Thurston and Seabury (1975) studied trap morphology of *Utricularia gibba* (= *Utricularia biflora*) and proposed that mucilage-like substances composed the velum. However, they used only scanning electron microscopy (SEM) and periodic acid-Schiff (PAS) reaction.

Recently, the scientific team of Simon Poppinga (Westermeier et al., 2017) investigated the trap biomechanics in 19 *Utricularia* species in order to determine any correlations between the life-forms, trapping mechanisms, and functional morphological traits. Although these authors observed a velum in the traps of *U. gibba* L. (sect. *Utricularia*) and *Utricularia resupinata* B.D.Greene ex Bigelow (sect. Lecticula), they questioned the occurrence of a velum in the traps of *Utricularia* species from the sections: *Polypompholyx* (*Utricularia multifida* R.Br.), *Pleiochasia* (*Utricularia uniflora* R.Br., *U. dichotoma*, *Utricularia menziesii* R.Br.), Nigrescentes (*Utricularia warburgii* K.I.Goebel), *Calpidisca* (*Utricularia welwitschii* Oliv., *Utricularia livida* E.Mey.), Stomoisia (*Utricularia cornuta* Michx.), *Oligocista* (*Utricularia prehensilis* E.Mey), *Foliosa* (*Utricularia calycifida* Benj., *Utricularia praelonga* A.St-Hil. and Girard), *Orchidioides* (*Utricularia alpina* Jacq., *Utricularia reniformis* A.St-Hil.), Setiscapella (*Utricularia flaccida* A.DC.), and Steyermarkia (*Utricularia aureomaculata* Steyerm).

In some species (*U. welwitschii*, *U. livida*, *U. cornuta*, *U. praelonga*, *U. reniformis*, *U. aureomaculata*), Westermeier et al. (2017) observed a structure at the outer zone of pavement epithelium, which they interpreted as an accumulation of mucilage. These authors proposed the absence of a velum in the members of sections *Polypompholyx, Pleiochasia, Nigrescentes, Calpidisca, Stomoisia, Oligocista, Foliosa, Orchidioides, Setiscapella*, and Steyermarkia. Thus, the results of Westermeier et al. (2017) are contradictory to the observations that were made by Lloyd (1942), Heide-Jørgensen (1989, 1991) and Reifenrath et al. (2006). The different opinions even concern the same species (i.e., *U. multifida*, *U. dichotoma*, and *U. reniformis*).

The main aim of this study was to test whether a velum is a common feature in the traps of *Utricularia* across a wide range of species and to explore differences and commonalities in trap morphology between species, with a focus on the region at and near the velum. Due to the fact that most authors consider the velum to be well developed in the trap of *U. vulgaris*, the *Utricularia reflexa* Oliv. (sect. *Utricularia*) was selected as the reference to other species.

Additionally, the general structure of the pavement epithelium among the species from various sections was studied and discussed.

## Materials and Methods

### Plant Material

The list of examined species with infra-generic classification in *Utricularia* was presented in Table 1. The plant material was obtained from the Botanic Garden of Jagiellonian University in Kraków (Poland), the collection of Kamil Pásek (Ostrava, Czech Republic, http://www.bestcarnivorousplants.net/), the collection of the Institute of Botany of the Czech Academy of Sciences at Treboň (Czech Republic), the collection of Dr. Corin Gardiner (New Zealand), and the collection of Mateusz Wrazidło (Poland). Approximately 10 or more traps were examined for each species.

**Table 1:** The list of examined species with infra-generic classification in *Utricularia*. according Taylor (1989) and Silva et al. (2018).

Subgenus	Section	Species
*Polypompholyx*	*Pleiochasia*	*U. novae-zelandiae* Hook.f. *U. volubilis* R.Br. *U. dichotoma* Labill. *U. tubulata* F.Muell.
	*Lasiocaules*	*U. uniflora* R.Br.
*Bivalvaria*	*Calpidisca*	*U. sandersonii* Oliv. *U. livida* E.Mey.
	*Oligocista*	*U. prehensilis* E.Mey.
*Utricularia*	*Foliosa*	*U. calycifida* Benj. *U. tricolor* A.St.-Hil.
	*Orchidioides*	*U. alpina* Jacq. *U. nelumbifolia* Gardner *U. humboldtii* R.H.Schomb.
	*Utricularia*	*U. reflexa* Oliv.
	*Vesiculina*	*U. purpurea* Walter

### Methods

The traps were examined using light microscopy (LM), scanning electron microscopy (SEM), and transmission electron microscopy (TEM) as follows. The traps were fixed in a mixture of 2.5% glutaraldehyde with 2.5% formaldehyde in a 0.05M cacodylate buffer (Sigma; pH 7.2) overnight or for several days, washed three times in a 0.1 M sodium cacodylate buffer and post-fixed in a 1% osmium tetroxide solution at room temperature for 1.5 h. These were followed by dehydration using a graded ethanol series, infiltration and embedding using an epoxy embedding medium kit (Fluka). Following polymerization at 60°C, sections were cut at 70 nm for the transmission electron microscopy (TEM) using a Leica ultracut UCT ultramicrotome, stained with uranyl acetate and lead citrate (Reynolds, 1963) and examined using a Hitachi H500 transmission electron microscope at an accelerating voltage of 75 kV.

Semi-thin sections (0.9–1.0 μm thick) were prepared for LM and stained for general histology using aqueous methylene blue/azure II (MB/AII) for 1–2 min (Humphrey and Pittman, 1974) and examined using an Olympus BX60 light microscope. Additionally, fresh, non-fixed traps were cut and examined using an Olympus BX60 light microscope. The periodic acid-Schiff (PAS) reaction was also used to reveal the presence of insoluble polysaccharides, and Sudan Black B (SBB) was used to detect the presence of lipids and cuticle material (Jensen, 1962). Staining for total proteins was performed using mercuric bromophenol blue (Mazia et al., 1953). For the SEM, the representative floral parts were fixed (as above or in ethanol in the case of *U. tubulata*) and later dehydrated and subjected to critical-point drying using liquid CO_2_. They were then sputter-coated with gold and examined at an accelerating voltage of 20 kV using a Hitachi S-4,700 scanning electron microscope (Hitachi, Tokyo, Japan), which is housed in the Institute of Geological Sciences, Jagiellonian University in Kraków).

## Results

### Section *Pleiochasia* (*U. novae-zelandiae*, *U. volubilis*, *U. dichotoma*, and *U. tubulata*)

**Figure 2:**
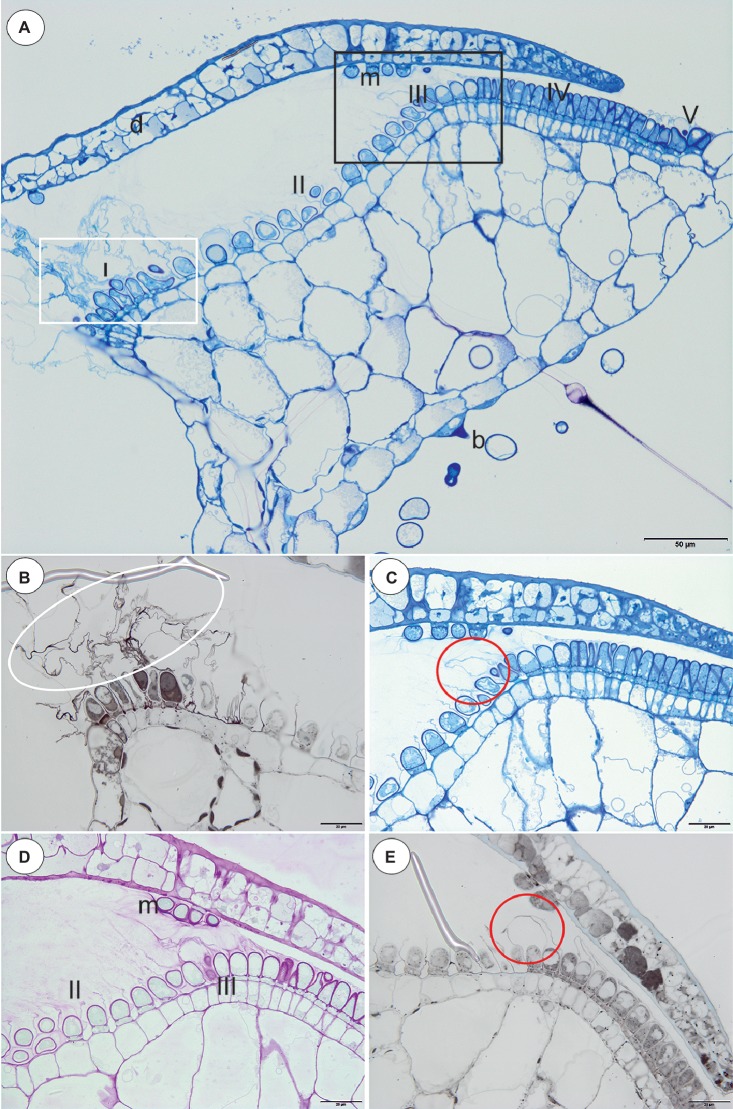
Structure and cytochemistry of the pavement epithelium of *Utricularia dichotoma*. **(A)** A semi-thin longitudinal section of the threshold showing the zones of the pavement epithelium: zone 1 (I) with an outer velum, zone 2 (II), zone 3 (III) with an inner velum, zone 4 (IV), zone 5 with mucilage trichomes (V), door (d), door mucilage trichomes (m), bifids (b); the sub-region marked by white box was shown in higher magnification at **(B)**, the sub-region marked by black box was shown in higher magnification at **(C,D)**; scale bar = 50 μm. **(B)** A semi-thin longitudinal section of the threshold of the external part showing the trichomes that produce the outer velum (white ellipse). The lipid stain SBB was absorbed by the cuticles; scale bar = 20 μm. **(C)** A semi-thin longitudinal section of part of the threshold showing zone 3 of the pavement epithelium with the trichomes that produce the inner velum (circle); scale bar = 20 μm. **(D)** The same as in C but after the PAS reaction, note the intensive mucilage production in zones 1 (II) and 3 (III) of the pavement epithelium, and also the mucilage trichomes on the surface of the door (m); scale bar = 20 μm. **(E)** A semi-thin longitudinal section of the part of the threshold showing zone 3 of the pavement epithelium with the trichomes that produce the inner velum (circle). The lipid stain SBB was absorbed by the cuticles; scale bar = 20 μm.

**Figure 3:**
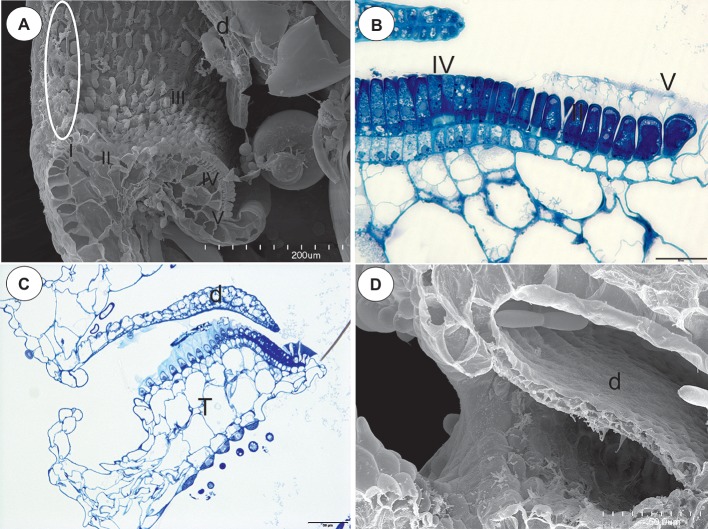
The structure of the pavement epithelium of the *Utricularia* species; sections *Pleiochasia* and *Lasiocaules*. **(A)** A longitudinal section of a *Utricularia tubulata* trap entrance, note the outer velum (white ellipse) and the zones of the pavement epithelium, door (d); scale bar = 200 μm. **(B)** *U. volubilis* A semi-thin longitudinal section of part of the threshold showing zone 4 (IV) and zone 5 with mucilage trichomes (V); scale bar = 20 μm. **(C)** A longitudinal section of a *Utricularia uniflora* trap entrance, threshold (T), door (d); scale bar = 50 μm. **(D)** SEM image of a longitudinal section of a *Utricularia uniflora* trap entrance, note the lack of an outer velum, door (d); scale bar = 50 μm.

In all of the species that were examined, most of the glandular trichomes in the exterior pavement epithelium produced the cuticle that formed the velum (zone 1; Figures 1B–F, 3A). According to the terminology of Lloyd (1942), this velum is the “outer velum.” This velum was also formed by the cuticles of the terminal cells of the trichomes that occurred at the front of the door. The velum was honeycomb-shaped (Figure 1E). This outer velum formed a complete ring (Figure 1F). The cuticles that formed the velum were stained with Sudan Black B (Figure 2B). In the next zone of the pavement epithelium (zone 2), there were trichomes with terminal cells, which did not touch the terminal cells of the neighboring trichomes (only their cuticles were connected). These trichomes produced mucilage (Figures 2C,D). In zone 3, the terminal cell of the trichomes produced mucilage and had prominent ruptured cuticles that formed the “inner velum” (Figures 1B, 2A). The cuticles that formed the inner velum were stained with Sudan Black B (Figure 2E). When the door was closed (set position), the mucilage and cuticles from the pavement epithelium trichomes touched the surface of the door (Figures 2A,C–E). Zone 4 was formed by trichomes that were closely packed, and therefore, their terminal cells touched each other (Figure 2A). There were mucilage trichomes in zone 5 that were less closely packed compared to the trichomes from zone 4 (Figure 2A). This difference was very well visible in the traps of *U. volubilis* (Figure 3B) and *U. dichotoma* but was less prominent in the *U. novae-zelandiae* traps.

### Section *Lasiocaules* (*U. uniflora*)

**Figure 4:**
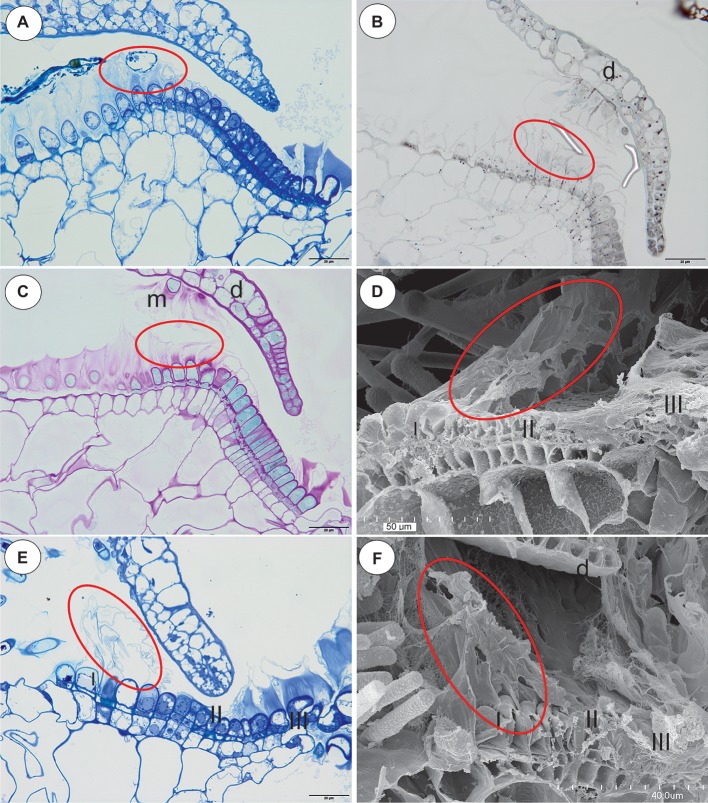
The structure and cytochemistry of the pavement epithelium of *Utricularia uniflora*, the structure of the pavement epithelium of the *Utricularia* species; section *Calpidisca*. **(A)** A longitudinal section of the pavement epithelium of *Utricularia uniflora*, note the velum (ellipse); scale bar = 20 μm. **(B)** *Utricularia uniflora*, a semi-thin longitudinal section of the threshold of the external part showing the trichomes that produce the velum. The lipid stain SBB was absorbed by the cuticles (ellipse); scale bar = 20 μm. **(C)** PAS reaction, note the intensive production of mucilage in zones I. II and IV of the pavement epithelium, and also the mucilage trichomes on the surface of the door (m), door (d); scale bar = 20 μm. **(D)** SEM image of a longitudinal section of a *Utricularia sandersonii* trap entrance, note the external zone (I) in which the trichomes produce velum (ellipse), the middle zone (II) and the internal zone (III) with the mucilage trichomes; scale bar = 50 μm. **(E)** A semi-thin longitudinal section of the pavement epithelium of *Utricularia sandersonii*, note the three zones of the pavement epithelium (I, II, III), the velum (ellipse); scale bar = 20 μm. **(F)** SEM image of a longitudinal section of *Utricularia livida*, note the external zone (I) in which the trichomes produce the velum (ellipse), the middle zone (II) and the internal zone (III) with the mucilage trichomes, door (d); scale bar = 50 μm.

The species in this section had no “outer velum.” In the exterior part of the pavement epithelium, there were glandular trichomes with terminal cells, which did not touch the terminal cells of the neighboring trichomes, only their ruptured cuticles were connected (zone 1, this zone corresponded to zone 2 in the section *Pleiochasia*) (Figures 3C,D). The terminal cells of these trichomes had ruptured cuticles (Figure 4A). In the next zone of the pavement epithelium–the terminal cells of the trichomes produced mucilage and had prominent ruptured cuticles (zone 2) (Figure 4A). The cuticles of the neighboring trichomes were connected and formed the velum, which stained with Sudan Black B (Figure 4B). When the door was closed (set position), the mucilage and cuticles from the pavement epithelium trichomes touched the surface of the door (Figures 4B,C). In the interior part of the pavement epithelium (curved part of threshold), the trichomes were closely packed, and therefore their terminal cells touched each other (zone 3). These trichomes did not have a very prominent ruptured cuticle in the terminal cells. The trichomes that were in the most interior part of the pavement epithelium (zone 4) produced a large amount of mucilage, which was well visible after using the aqueous methylene blue/azure II stain (Figure 4A) and after the PAS reaction (Figure 4C).

### Section *Calpidisca* (*U. sandersonii*, *U. livida*)

There were three distinct zones in the pavement epithelium (Figures 4D–F): an external zone (1) in which the trichomes produced the velum (about four rows of trichomes), a middle zone (2) with trichomes that had flattened terminal cells, and an internal zone (3) that had mucilage trichomes. The trichomes of the internal zone produced a large amount of mucilage, which was well visible in both the semi-thin sections (Figure 4E) and the SEM picture (Figures 4D,F). All of the trichomes of the pavement epithelium had a ruptured cuticle of terminal cells. The velum formed a valve that touched the free edge of the trap door (Figure 4E). In the front of the pavement epithelium, there were long-stalked glands, which produced a large amount of mucilage (Figures 4D–F).

### Section *Oligocista* (*U. prehensilis*) and Section *Foliosa* (*U. calycifida*, *Utricularia tricolor*)

**Figure 5:**
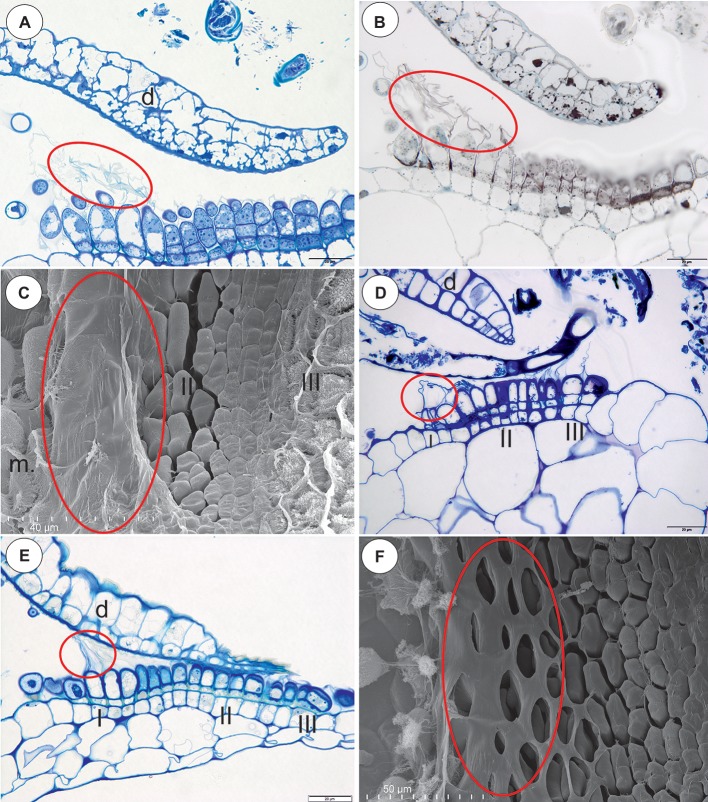
Structure of the pavement epithelium and velum of *Utricularia* species; section *Oligocista* (*U. prehensilis*) and section *Foliosa* (*U. calycifida*, *U. tricolor*). **(A)** A semi-thin longitudinal section of the pavement epithelium of *Utricularia prehensilis*; the velum (ellipse); scale bar = 20 μm. **(B)** *U. prehensilis*, a semi-thin longitudinal section of the threshold of the external part showing the trichomes that produce the velum (ellipse). The lipid stain SBB was absorbed by the cuticles and the lateral walls of the barrier cells; scale bar = 20 μm. **(C)** *Utricularia calycifida*, scanning electron microscope image of the pavement epithelium, note the velum that covers zone one (ellipse), the middle zone (II) and the internal zone (III) with the mucilage trichomes and the sessile mucilage trichomes at the front of the pavement epithelium (m); scale bar = 40 μm. **(D)** A semi-thin longitudinal section of the pavement epithelium of *Utricularia calycifida*, note the three zones of the pavement epithelium (I, II, III), the velum (circle); scale bar = 20 μm. **(E)** A semi-thin longitudinal section of the pavement epithelium of *Utricularia tricolor*, note the three zones of the pavement epithelium (I, II, III), the velum (circle); scale bar = 20 μm. **(F)** *Utricularia prehensilis*, SEM image of the pavement epithelium, note the honeycomb-like velum (ellipse); scale bar = 50 μm.

In the pavement epithelium, there were three distinct zones (Figures 5A–E): an external zone in which the trichomes produced velum (Figures 5A,B), a middle zone with trichomes that had flattened terminal cells and an internal zone with mucilage trichomes. The cuticles that formed the velum were stained with Sudan Black B (Figure 5B). The velum that was formed by the glands from the external zone had a membrane-like structure (Figure 5C) or a honeycomb-like structure (Figure 5F), and the apexes of the glandular cells were visible. In the front of the pavement epithelium, there were sessile glands that produced a large amount of mucilage (Figures 5C,F).

### Section *Orchidioides* (*U. alpina*, *U. nelumbifolia*, *U. humboldtii*)

**Figure 6:**
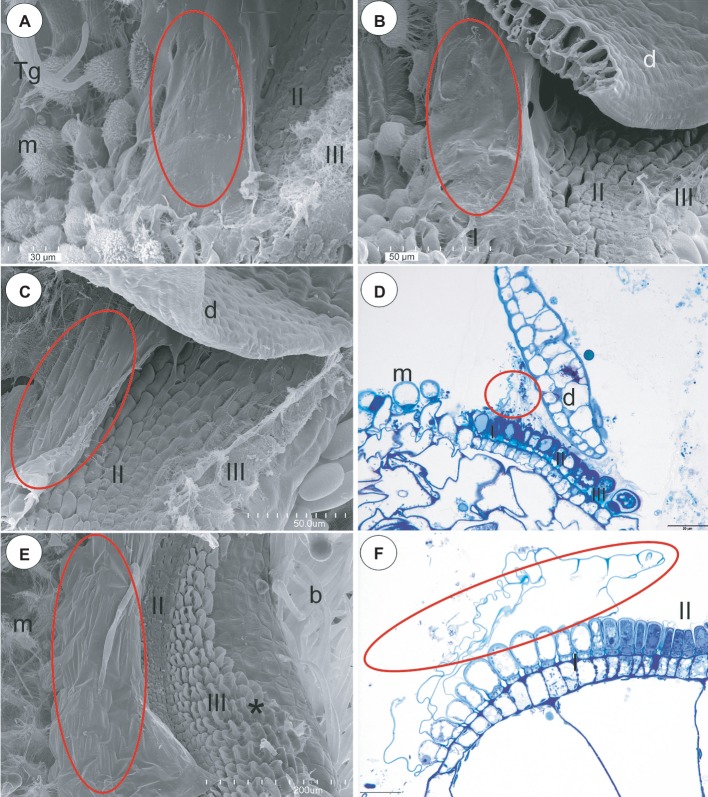
Structure of the pavement epithelium and velum of *Utricularia* species; section *Orchidioides* (*Utricularia alpina*, *Utricularia nelumbifolia*, *Utricularia humboldtii*) and section *Utricularia* (*U. reflexa*). **(A)** *Utricularia alpina*, SEM image of the pavement epithelium, note the velum that covers zone one (ellipse), the middle zone (II) and the internal zone (III) with the mucilage trichomes and the sessile mucilage trichomes at the front of the pavement epithelium (m), trigger bristles (Tg); scale bar = 30 μm. **(B)** *Utricularia humboldtii*, SEM image of the pavement epithelium, note the velum (ellipse), zone one (I), the middle zone (II) and the internal zone (III) with the mucilage trichomes and the sessile mucilage trichomes at the front of the pavement epithelium (m), door (d); scale bar = 50 μm. **(C)** *Utricularia nelumbifolia*, SEM image of the pavement epithelium, note the velum that covers zone one (ellipse), the middle zone (II) and the internal zone (III) with the mucilage trichomes, trap door (d); scale bar = 50 μm. **(D)** *Utricularia nelumbifolia*, a semi-thin longitudinal section of the pavement epithelium, note the three zones of the pavement epithelium (I, II, III), the velum (circle), trap door (d), the mucilage trichomes (m); scale bar = 20 μm. **(E)** *Utricularia reflexa,* SEM image of the pavement epithelium, note the velum (ellipse), the middle zone (II) and the internal zone (III) with the mucilage trichomes (asterisk) and the mucilage trichomes at the front of the pavement epithelium (m), bifids (b); scale bar = 200 μm. **(F)** *Utricularia reflexa*, a semi-thin longitudinal section of part of the pavement epithelium, note the zones of pavement epithelium (I, II), the velum (ellipse); scale bar = 20 μm.

In the pavement epithelium, there were three distinct zones: an external zone in which the trichomes produced the velum (zone 1), a middle zone with trichomes that had flattened terminal cells and an internal zone that had mucilage trichomes (Figures 6A–D). The velum was formed by the cuticles of the most external pavement trichomes and connected with the cuticles of the more internal trichomes. Thus, a balloon-like structure was formed (Figures 6A–C). In *U. nelumbifolia*, the velum was colonized by bacteria (Figure 6D). In the front of the pavement epithelium, there were mucilage trichomes (Figures 6A–D).

### Section *Utricularia* (*U. reflexa*)

**Figure 7:**
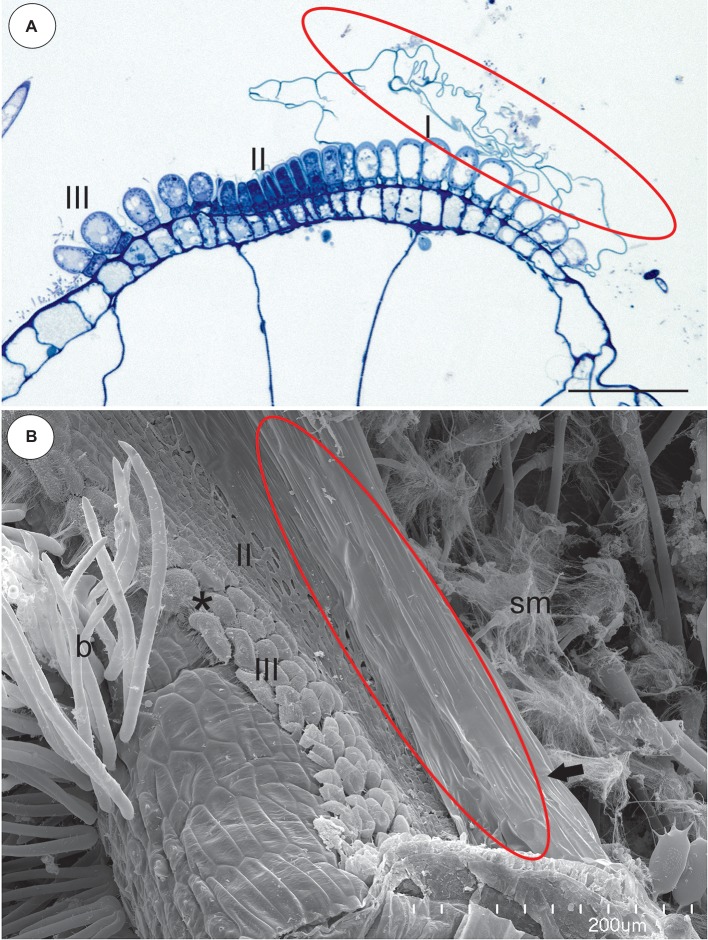
Structure of the pavement epithelium and velum of *Utricularia reflexa*. **(A)** A semi-thin longitudinal section of the pavement epithelium, note all of the zones of the pavement epithelium (I, II, III), the velum (ellipse); scale bar = 50 μm. **(B)** A SEM image of the pavement epithelium, note the velum (ellipse), the middle zone (II) and the internal zone (III) with the mucilage trichomes (asterisk) and the stalked mucilage trichomes at the front of the pavement epithelium (sm), bifids (b); scale bar = 200 μm.

**Figure 8:**
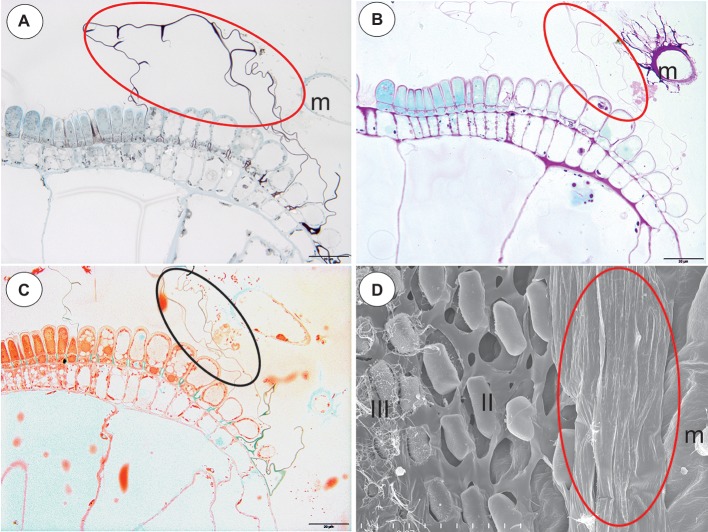
Staining of the pavement epithelium and velum of *Utricularia reflexa* and *U. purpurea*. **(A)** *Utricularia reflexa*, a semi-thin longitudinal section of the threshold of the external part showing the trichomes that produce the velum (ellipse). The lipid stain SBB was absorbed by the cuticles and lateral walls of the barrier cells; scale bar = 20 μm. **(B)** *Utricularia reflexa*, PAS reaction, note the pink colouration of the velum (ellipse) and the very intensive mucilage production by the stalked trichomes (m); scale bar = 20 μm. **(C)** *Utricularia reflexa*, staining for total proteins noted no proteins in the velum (ellipse) but a positive staining of the bacteria and cytoplasm of the cells of the pavement epithelium trichomes; scale bar = 20 μm. **(D)** *Utricularia purpurea*, a SEM image of the pavement epithelium, note the velum (ellipse), the middle zone (II) and the internal zone (III) with the mucilage trichomes and the sessile mucilage trichomes at the front of the pavement epithelium (m); scale bar = 50 μm.

In the pavement epithelium, there were three distinct zones: an external zone (1), a middle zone (2), and an internal zone that had mucilage glands (3) (Figures 6E,F and Figures 7A,B). The velum was primarily formed by the cuticles of the external zone trichomes and was a balloon-like structure (Figure 6F). It covered the external zone in a fixed material (Figures 6E,F and Figures 7A,B). The velum continued with the cuticles of the middle zone trichomes–this part looked like a honeycomb (Figure 7B). The cuticles that formed the velum stained very deeply with Sudan Black B (Figure 8A), but only weakly after the PAS reaction (Figure 8B). Staining for total proteins did not reveal any proteins in the velum (only bacteria were stained) (Figure 8C). In the front of the pavement epithelium, there were long-stalked pyriform glands that produced a large amount of mucilage (Figures 7B, 8B).

### Section *Vesiculina* (*U. purpurea*)

The pavement epithelium was smaller than that in *U. reflexa*. In the pavement epithelium, there were three distinct zones: an external zone in which the trichomes produced velum, a middle, and an internal zone with mucilage trichomes (Figure 8D). The cuticles of the trichomes from both the middle and internal zones were connected and formed a honeycomb-like structure, and therefore, the terminal trichome cells were visible (Figure 8D).

### Ultrastructure Data (*U. dichotoma*, *U. uniflora*, *U. prehensilis*, *U. nelumbifolia*, *U. reflexa*)

**Figure 9:**
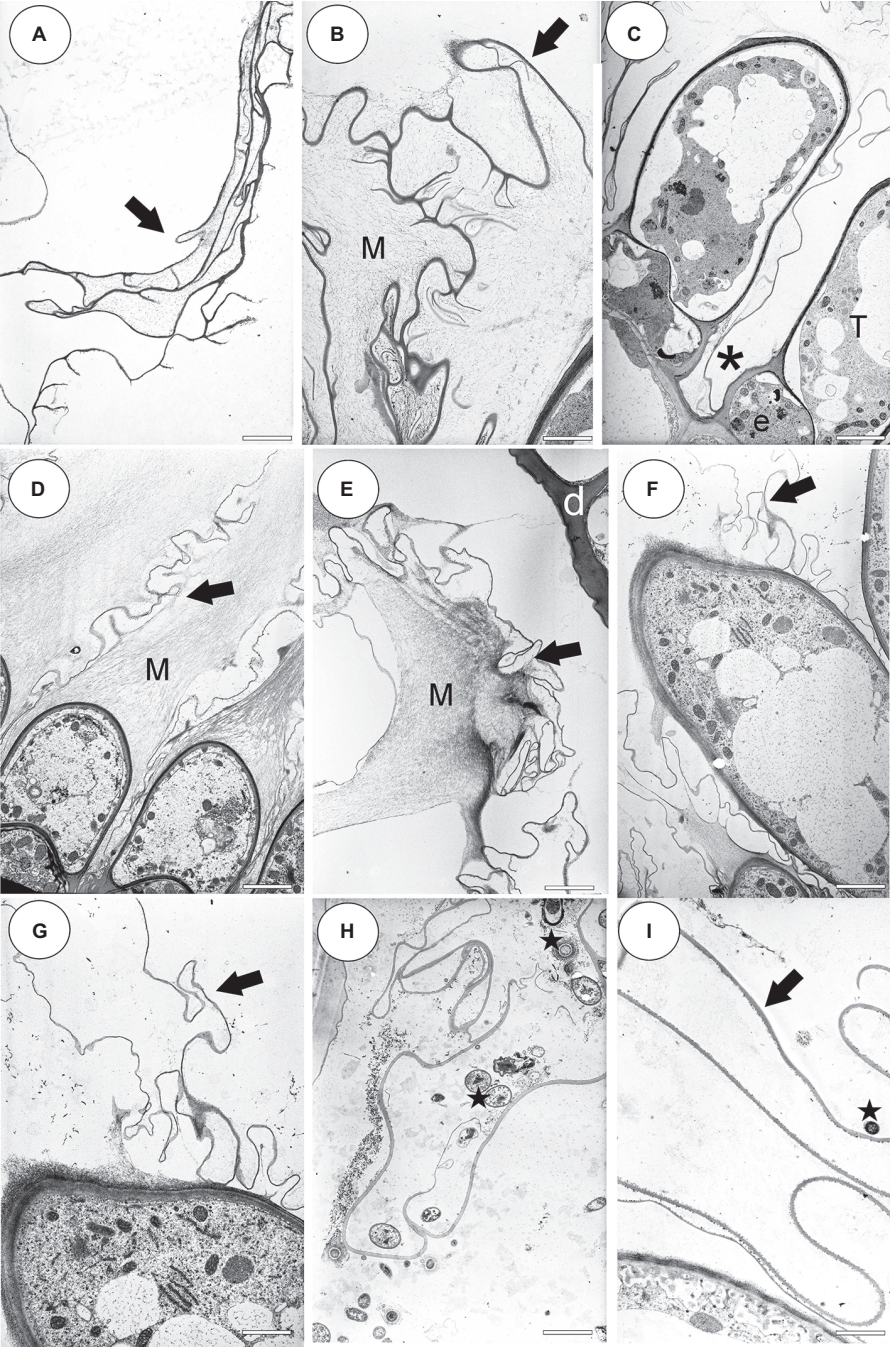
Ultrastructure of the velum and pavement epithelium trichomes. **(A)** An external velum (arrow) ultrastructure of *Utricularia dichotoma*; this part of the velum was formed by the cuticles of the threshold trichomes; scale bar = 1.92 μm. **(B)** External velum (arrow) ultrastructure of *Utricularia dichotoma*, note the mucilage (M); scale bar = 1.92 μm. **(C)** Ultrastructure of the pavement epithelium trichomes of *Utricularia dichotoma*, note that the velum was formed by the cuticles (asterisk) of the terminal cells (T) and also the middle (barrier) cells of the trichomes (e); scale bar = 3.66 μm. **(D)** Ultrastructure of the velum (arrow) and pavement epithelium trichomes of *Utricularia uniflora*, note the mucilage (M); scale bar = 4 μm. **(E)** Velum (arrow) ultrastructure of *Utricularia uniflora*, note the mucilage (M); scale bar = 3.14 μm. **(F,G)** Velum (arrow) formation in *Utricularia prehensilis*, note that mucilage was also secreted; scale bar = 3.6 μm and bar = 2.55 μm. **(H)** Velum (arrow) ultrastructure of *Utricularia nelumbifolia*, note the bacteria (star); scale bar = 3 μm. **(I)** Velum (arrow) ultrastructure of *Utricularia reflexa*, note the bacteria (star); scale bar = 2.55 μm.

The velum consisted of cuticles (Figures 9A–I) that were formed by the terminal cells and also the middle (barrier) cells of the trichomes (Figure 9C). There was mucilage that had the character of fine fibers (i.e., *U. dichotoma*, *U. uniflora* Figures 9B,D,E) or granules that were on the surface of the velum (i.e., *U. reflexa*. Figure 9I) in some of the cuticles. A large amount of mucilage was observed in *U. dichotoma* and *U. uniflora* (Figures 9B,D,E). The surface of the velum was colonized by bacteria in some of the species (Figures 9H,I).

## Discussion

Regarding the occurrence of velum, our results support the observations of Lloyd (1932b, 1942, see Supplementary Material 1), Heide-Jørgensen (1989, 1991), Richter (1990) and Reifenrath et al. (2006). Proper techniques and various methods were essential to show that there exists a wide range of velum morphologies across *Utricularia* species. It should be highlighted that the method of preparing the specimen may affect some features that they cannot be clearly identified. For example, absence of a velum might be a methodological artifact because only SEM was used and this did not allow distinction between mucilage and cuticles. Even Lloyd wrote “*The presence of this veil has been hitherto overlooked; indeed, I have overlooked it myself for years. It is only with great care that sections can be cut (of fresh material, of course) without tearing it away.”* (Lloyd, 1929, p. 93). So, light microscopy, cytochemical staining and TEM allow good visibility of important trap features (allowed to distinguish the mucilage material from the cuticles) and clearly increase our current understanding of trap anatomy, such as the fact that there exists a wide range of velum morphologies across *Utricularia* species. Using good-quality thin sections, staining and TEM, it is clearly evident that both a velum (which is consisted of cuticle) and mucilage are produced by the glandular pavement epithelium trichomes in the *Utricularia* species from all three subgenera: *Polypompholyx* (sections *Pleiochasia* and *Lasiocaules*)*, Bivalvaria*, and *Utricularia*.

However, it should be highlighted that some authors e.g., Broussaud and Vintejoux (1982) were not completely sure about their definition/interpretation of the notion “velum” as compared to Lloyd’s usage of the term “velum.” Probably, Westermeier et al. (2017) preferred a narrow definition of velum (velum which occurs in *Utricularia vulgaris* trap types). Fineran (1985) hypothesized that the pavement epithelium and velum in *U. monanthos* (section *Pleiochasia*) were different compared to those in the trap type of *U. vulgaris*. Also Richter (1990) found differences with respect of the velum morphology between *Utricularia* cf. *praelonga* and *U. dichotoma*.

According to Lloyd (1932b, 1942) and this study in *Utricularia* species from section *Pleiochasia* (Lloyd’s *Utricularia hookeri* trap type), there is a double velum: an outer and inner velum. The outer velum is not only formed of the cuticles of trichomes of the pavement epithelium but also of the cuticles of the other trichomes of the threshold. Interestingly, in the *U. uniflora* trap, there is an absence of the threshold trichomes that form the outer velum. Jobson et al. (2017, 2018) classified this species to a new section *Lasiocaules*. It should be determined whether this character also occurs in other species from this section, which could be an important synapomorphy to add support to this monophyletic section.

After analyzing the velum structure in various *Utricularia* representatives, two main types of velum could be recognized; however, there are also transitional types. The first type, a velum with a balloon-like structure in which the apexes of the terminal cells are not visible (they are covered by the velum) in the part of the velum that is produced by the trichomes of the external part of the pavement epithelium. Most of the velum is formed by a continuous membrane that has no openings (Figure 7B). Outlines of individual fragments of the cuticles that form the velum are visible on the surface of the velum. This type of velum is a characteristic of the members of the section *Utricularia*. The velum in the section *Orchidioides* is very similar to the first type, but some apexes of the terminal cells may be visible in the part of the velum that is produced by the trichomes of the external part of the pavement epithelium (e.g., Figure 6A). In the second type, the velum structure is honeycomb shaped (Figure 1E). This type of velum is a characteristic of the members of the subg. *Polypompholyx*. This type structure of velum enables the production of a large amount of mucilage by the trichomes. Thus, a complex structure is formed (cuticles + mucilage). A modified honeycomb-like velum occurs in some of the *Utricularia* species of the subg. *Bivalvaria* (Figure 5F) but with less mucilage being produced by the trichomes from zone 1.

**Figure 10:**
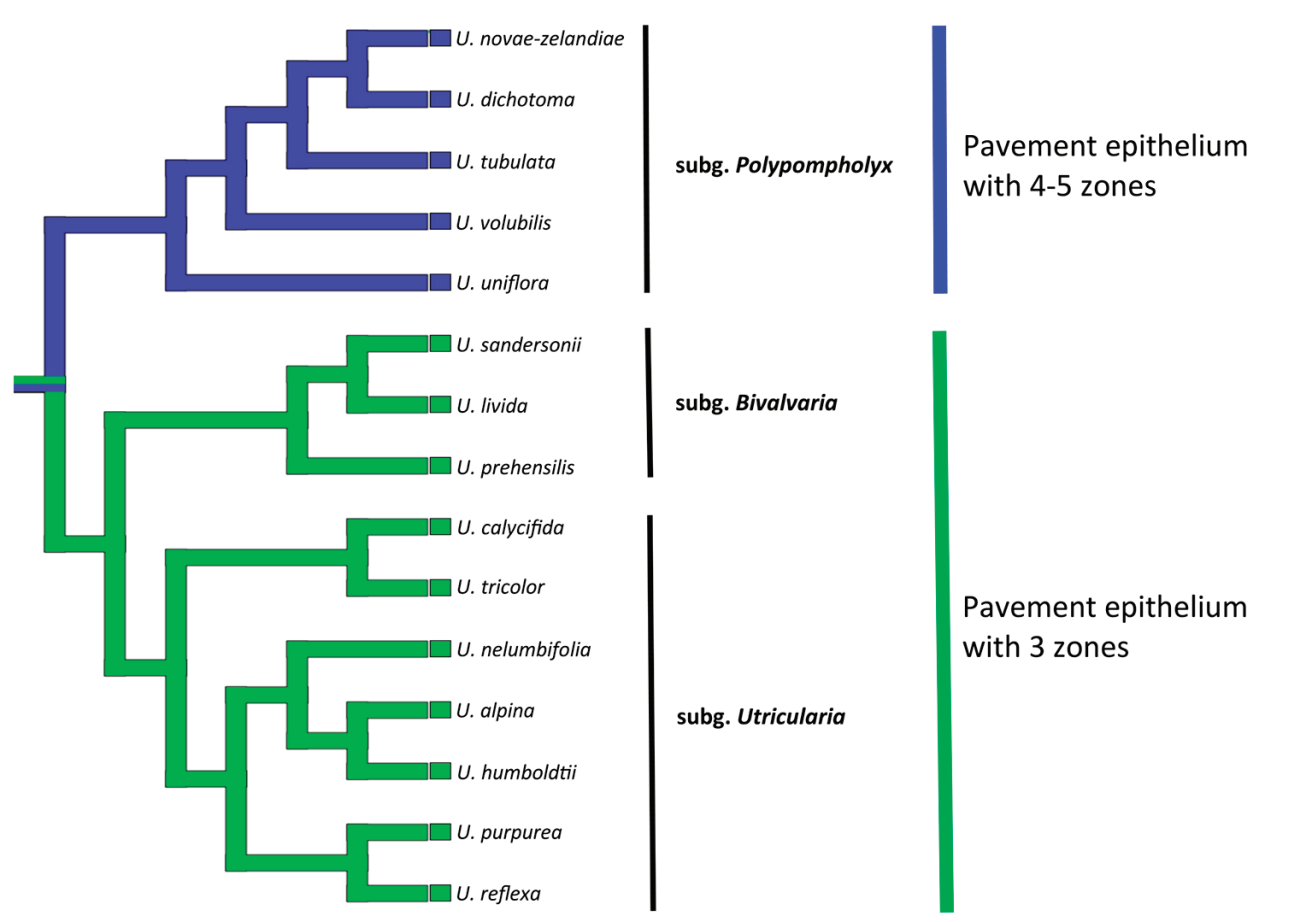
Phylogenetic hypothesis for the pavement epithelium (states: 3 zones or 4–5 zones) for *Utricularia* lineages. The topology was adapted from Jobson et al. (2017), Rodrigues et al. (2017), and Silva et al. (2018).

In the members of both sections *Pleiochasia* and *Lasiocaules*, the pavement epithelium had a more complicated structure (four to five zones) than in the members of the subgenera *Bivalvaria* and *Utricularia* in which three distinct zones occurred (an outer zone with the main zone of velum production, a middle zone, and an internal zone that had mucilage trichomes). Even in *U. purpurea*, in which the threshold was smaller (“rudimentary threshold” according to Reifenrath et al., 2006), the pavement epithelium consisted of three functional zones and there was a velum. Broussaud and Vintejoux (1982) recognized four zones in the pavement epithelium in the traps of *U. vulgaris*, *U. australis*; however, they considered ultrastructure details, e.g., the occurrence of cell wall ingrowths, which have not been not studied or considered in this research. According to a phylogenetic perspective, the pavement epithelium with four to five functional zones supports the clade of subg. *Polypompholyx*, while that with three zones occurs in the *Bivalvaria* + *Utricularia* clade (Figure 10). Thus, this character can be important for the phylogeny of the *Utricularia* lineages, bringing also support to the infrageneric classification. But, a wider sampling of species will be important to figure out the plesiomorphic state, thus to propose a hypothesis whether the three-zone epithelium is the ancestral state or originated by a reduction. The development of the velum deserves further detailed research, especially due to the fact that there is enormous diversity in *Utricularia* traps. Lloyd (1932b) recognized 15 main types of *Utricularia* traps that differ in various features such as the trap entrances, the occurrence of glandular trichomes and the trap vascular system (e.g., Lloyd, 1931, 1932a,b, 1942; Reifenrath et al., 2006; Płachno et al., 2017).

Westermeier et al. (2017) made important suggestion that the large amounts of mucilage in the entrance of traps with narrow-angled doors in the tubular trap entrances seal and fasten the oblique door. Therefore, the door in the traps of non-aquatic *Utricularia* is less susceptible to mechanical disturbances, which might be an advantage when the traps are not constantly surrounded by water. The production of mucilage by the trichomes of the pavement epithelium was found in all of the species that were examined here (both aquatic and terrestrial species). Mucilage was also produced by the trichomes that were in front of the pavement epithelium in the *Utricularia* species of the subg. *Bivalvaria* (Figures 5C–F) and the subg. *Utricularia* (Figures 6A,B, 7B, 8B, Heide-Jørgensen, 1989; Juniper et al., 1989). So mucilage helps velum to seal the door tip against the threshold.

The velum was considered to be a structure that consisted of the cuticles of the terminal cells of the trichomes (Lloyd, 1942; Heide-Jørgensen, 1991). Here, it is shown that the velum is not only formed by the cuticles of the terminal cells but also by the cuticles of the middle (barrier) cells of the trichomes. So the Lloyd (1942) definition of term “velum” is: velum–the cuticle structure which is produced by cells of pavement epithelium trichomes and forms membrane to help sealing the trap door. However, in TEM, we showed that cuticle membrane was covered by mucilage; thus, we propose a broad definition of velum as cuticle membranes covered by mucilage from the pavement epithelium trichomes touching the surface of the door in set position and also seals the trap door in resting reset state and, thus, it maintains the produced negative pressure necessary for trap firing and functioning.

## Conclusions

A velum occurs in the *Utricularia* traps regardless of the trap type or the habitat (aquatic, epiphytic, and terrestrial species). The results obtained here confirmed the observation of Francis Lloyd about the occurrence of a velum; however, we proposed broad definition of velum as cuticle membranes covered by mucilage. From a functional point of view, this definition is more useful and more reflects complexity of this structure. The finding of distinct velum structures in traps in all 15 *Utricularia* species studied supports the view that all these generic sections do produce negative pressure inside their traps and are thus able to fire and capture prey.

## Data Availability

All datasets generated for this study are included in the manuscript and/or the Supplementary Files.

## Author Contributions

All authors contributed to the conception and design of the manuscript. BP conducted study, analysed data, and wrote the manuscript. All authors discussed the results and commented, corrected and approved the manuscript.

### Conflict of Interest Statement

The authors declare that the research was conducted in the absence of any commercial or financial relationships that could be construed as a potential conflict of interest.

## Supplementary Material

SUPPLEMENTARY FIGURE 1 Comparison of Lloyd’s *Utricularia* trap (1942) drawings with our documentation; the same structures are marked by eclipses. **(A)** Section of *Utricularia monanthos* trap entrance; the outer velum (red ellipse), the inner velum (green ellipse). **(B)** *Utricularia dichotoma*, a semi-thin longitudinal section of the threshold showing the outer velum (red ellipse), the inner velum (green ellipse); scale bar = 50 μm. **(C)** *Utricularia monanthos*, trap opening is guarded by the outer velum (red ellipse). **(D)** *Utricularia novae-zelandiae*, view of a trap entrance showing the ring of the velum (red circle); scale bar = 200 μm. **(E)** Transverse selection of the threshold showing velum (red ellipse) of *Utricularia vulgaris* trap type. **(F)** *Utricularia reflexa*, a semi-thin longitudinal section of the threshold showing velum (red ellipse); scale bar = 50 μm.

## References

[ref1] Adamec L. (2011a). The comparison of mechanically stimulated and spontaneous firings in traps of aquatic carnivorous *Utricularia* species. Aquat. Bot. 94, 44–49. 10.1016/j.aquabot.2010.09.004

[ref2] Adamec L. (2011b). Functional characteristics of traps of aquatic carnivorous *Utricularia* species. Aquat. Bot. 95, 226–233. 10.1016/j.aquabot.2011.07.001

[ref3] Adamec L. (2012). Firing and resetting characteristics of carnivorous *Utricularia reflexa* traps: physiological or only physical regulation of trap triggering? Phyton 52, 281–290.

[ref4] Alkhalaf I. A. Hübener T. Porembski S. (2009). Prey spectra of aquatic *Utricularia* species (*Lentibulariaceae*) in northeastern Germany: the role of planktonic algae. Flora - Morphol. Distrib. Funct. Ecol. Plants 204, 700–708. 10.1016/j.flora.2008.09.008

[ref5] Broussaud F. Vintejoux C. (1982). Etudes ultrastructurales et cytochimiques des tissus superficiels placés à l’entrée des urnes d’*Utricularia* (Lentibulariacées). Bull. Soc. Bot., Lettres Bot, France 129, 191–20l.

[ref6] Darnowski D. Bauer U. Méndez M. Horner J. Płachno B. J. (2018). “Prey selection and specialization by carnivorous plants” in Carnivorous plants: Physiology, ecology, and evolution. eds. Ellison A. M. Adamec L. (Oxford, UK: Oxford University Press), 285–293. ISBN: 978019877984

[ref7] Fineran B. A. (1985). Glandular trichomes in *Utricularia*: a review of their structure and function. Israel J. Bot. 34, 295–330.

[ref8] Heide-Jørgensen H. S. (1989). Kødædende planter 3. Nat. Verden 9, 337–357.

[ref9] Heide-Jørgensen H. S. (1991). Cuticle development and ultrastructure: evidence for a procuticle of high osmium affinity. Planta 183, 511–519. 10.1007/BF00194272, PMID: 24193844

[ref10] Humphrey C. Pittman G. (1974). A simple methylene blue-azure II-basic fuchsin for epoxy-embedded tissue sections. Stain. Technol. 49, 9–14. 4131626 10.3109/10520297409116929

[ref11] Jensen W. A. (1962). Botanical histochemistry–principles and practice. University of California, Berkeley: W. H. Freeman and Company.

[ref12] Jobson R. W. Baleeiro P. C. Guisande C. (2018). “Systematics and evolution of *Lentibulariaceae*: III. *Utricularia*” in Carnivorous plants: Physiology, ecology, and evolution. eds. Ellison A. M. Adamec L. (Oxford, UK: Oxford University Press), 89–104. ISBN: 978019877984

[ref13] Jobson R. W. Baleeiro P. C. Reut M. (2017). Molecular phylogeny of subgenus *Polypompholyx* (*Utricularia*; *Lentibulariaceae*) based on three plastid markers: diversification and proposal for a new section. Aust. Syst. Bot. 30, 259–278. 10.1071/SB17003

[ref14] Juniper B. E. Robins R. J. Joel D. M. (1989). The carnivorous plants. London: Academic.

[ref15] Lloyd F. E. (1929). The mechanism of the water tight door of the *Utricularia* trap. Plant Physiol. 4, 87–102. 10.1104/pp.4.1.87, PMID: 16652602 PMC440037

[ref16] Lloyd F. E. (1931). The range of structural and functional variation in the traps of *Utricularia*. Flora 25, 260–276.

[ref17] Lloyd F. E. (1932a). The door of *Utricularia*, an irritable mechanism. Can. J. Bot. 10, 780–786.

[ref18] Lloyd F. E. (1932b). The range of structural and functional variety in the traps of *Utricularia* and *Polypompholyx*. Flora 126, 303–328.

[ref19] Lloyd F. E. (1942). The carnivorous plants. Waltham, Massachusetts: Chronica Botanica Company, Donald Publishing.

[ref20] Mazia D. Brewer P. A. Alfert M. (1953). The cytochemical staining and measurement of protein with mercuric bromophenol blue. Biol. Bull. 104, 57–67. 10.2307/1538691

[ref21] Mette N. Wilbert N. Barthlott W. (2000). Food composition of aquatic bladderworts (*Utricularia*, *Lentibulariaceae*) in various habitats. Beitr. Biol. Pflanzen 72, 1–13.

[ref22] Płachno B. J. Adamec L. Kamińska I. (2015). Relationship between trap anatomy and function in Australian carnivorous bladderworts (*Utricularia*) of the subgenus *Polypompholyx*. Aquat. Bot. 120, 290–296. 10.1016/j.aquabot.2014.09.008

[ref23] Płachno B. J. Kamińska I. Adamec L. Świątek P. (2017). Vascular tissue in traps of Australian carnivorous bladderworts (*Utricularia*) of the subgenus *Polypompholyx*. Aquat. Bot. 142, 25–31. 10.1016/j.aquabot.2017.06.003

[ref24] Płachno B. J. Łukaszek M. Wołowski K. Adamec L. Stolarczyk P. (2012). Aging of *Utricularia* traps and variability of microorganisms associated with that microhabitat. Aquat. Bot. 97, 44–48. 10.1016/j.aquabot.2011.11.003

[ref25] Płachno B. J. Muravnik L. E. (2018). “Chapter 13. Functional anatomy of carnivorous traps” in Carnivorous plants: Physiology, ecology, and evolution. eds. Ellison A. M. Adamec L. (Oxford, UK: Oxford University Press), 167–179. ISBN: 978019877984

[ref26] Płachno B. J. Wołowski K. Fleischmann F. Lowrie A. Łukaszek M. (2014). Algae and prey associated with traps of the Australian carnivorous plant *Utricularia* volubilis (*Lentibulariaceae*, *Utricularia* subgenus *Polypompholyx*) in natural habitat and in cultivation. Aust. J. Bot. 62, 528–536. 10.1071/BT14176

[ref27] Poppinga S. Daber L. E. Westermeier A. S. Kruppert S. Horstmann M. Tollrian R. (2017). Biomechanical analysis of prey capture in the carnivorous Southern bladderwort (*Utricularia australis*). Sci. Rep. 7:1776. 10.1038/s41598-017-01954-3 28496168 PMC5431978

[ref28] Poppinga S. Masselter T. Speck T. (2013). Faster than their prey: new insights into the rapid movements of active carnivorous plants traps. BioEssays 35, 649–657. 10.1002/bies.201200175, PMID: 23613360

[ref29] Poppinga S. Weisskopf C. Westermeier A. S. Masselter T. Speck T. (2016). Fastest predators in the plant kingdom: functional morphology and biomechanics of suction traps found in the largest genus of carnivorous plants. AoB Plants 8plv140. 10.1093/aobpla/plv140, PMID: 26602984 PMC4717191

[ref30] Reifenrath K. Theisen I. Schnitzler J. Porembski S. Barthlott W. (2006). Trap architecture in carnivorous *Utricularia* (*Lentibulariaceae*). Flora 201, 597–605. 10.1016/j.flora.2005.12.004

[ref31] Reynolds E. S. (1963). The use of lead citrate at high pH as an electronopaque stain for electron microscopy. J. Cell Biol. 17, 208–212. 10.1083/jcb.17.1.208, PMID: 13986422 PMC2106263

[ref32] Richter U. (1990). Die Fangblasen von *Utricularia* cf. *praelonga* St.Hil. und *Utricularia* dichotoma Lab.–eine rasterelektronische Untersuchung. Flora 184, 21–30. 10.1016/S0367-2530(17)31582-7

[ref33] Rodrigues F. G. Marulanda N. F. Silva S. R. Płachno B. J. Adamec L. Miranda V. F. O. (2017). Phylogeny of the “orchid-like” bladderworts (gen. *Utricularia* sect. *Orchidioides* and Iperua: *Lentibulariaceae*) with remarks on the stolon-tuber system. Ann. Bot. 120, 709–723. 10.1093/aob/mcx056, PMID: 28673037 PMC5691873

[ref34] Silva S. R. Gibson R. Adamec L. Domínguez Y. Miranda V. F. O. (2018). Molecular phylogeny of bladderworts: a wide approach of *Utricularia* (*Lentibulariaceae*) species relationships based on six plastidial and nuclear DNA sequences. Mol. Phylogenet. Evol. 118, 244–264. 10.1016/j.ympev.2017.10.010, PMID: 29054811

[ref35] Sirová D. Bárta J. Borovec J. Vrba J. (2018). “The *Utricularia*-associated microbiome: composition, function, and ecology” in Carnivorous plants: Physiology, ecology, and evolution. eds. Ellison A. M. Adamec L. (Oxford, UK: Oxford University Press), 349–358. ISBN: 9780198779841

[ref36] Sirová D. Borovec J. Černá B. Rejmánková E. Adamec L. Vrba J. (2009). Microbial community development in the traps of aquatic *Utricularia* species. Aquat. Bot. 90, 129–136. 10.1016/j.aquabot.2008.07.007

[ref170] Taylor P. (1989). The Genus Utricularia: A Taxonomic Monograph. Kew Bulletin, Additional Series, XIV.

[ref37] Thurston E. L. Seabury F. (1975). A scanning electron microscopic study of the utricle trichomes in *Utricularia* biflora Lam. Bot. Gaz. 136, 87–93. 10.1086/336786

[ref38] Vincent O. Marmottant P. (2011). Carnivorous *Utricularia*: the buckling scenario. Plant Signal. Behav. 6, 1752–1754. 10.4161/psb.6.11.17804 22067995 PMC3329349

[ref39] Vincent O. Roditchev I. Marmottant P. (2011a). Spontaneous firings of carnivorous aquatic *Utricularia* traps: temporal patterns and mechanical oscillations. PLoS One 6:e20205. 10.1371/journal.pone.0020205 21647417 PMC3103537

[ref40] Vincent O. Weißkopf C. Poppinga S. Masselter T. Speck T. Joyeux M. (2011b). Ultra-fast underwater suction traps. Proc. R. Soc. B Biol. Sci. 278, 2909–2914. 10.1098/rspb.2010.2292 PMC3151700 21325323

[ref41] Westermeier A. S. Fleischmann A. Müller K. Schäferhoff B. Rubach C. Speck T.. (2017). Trap diversity and character evolution in carnivorous bladderworts (*Utricularia*, *Lentibulariaceae*). Sci. Rep. 7:12052. 10.1038/s41598-017-12324-4, PMID: 28935893 PMC5608911

