# Supplementary figures and images for: The Structure and Occurrence of a Velum in Utricularia Traps (Lentibulariaceae)
Source: Front Plant Sci. 2019 Mar 22;10:302. doi: 10.3389/fpls.2019.00302 (PMC6454230; doi:10.3389/fpls.2019.00302)

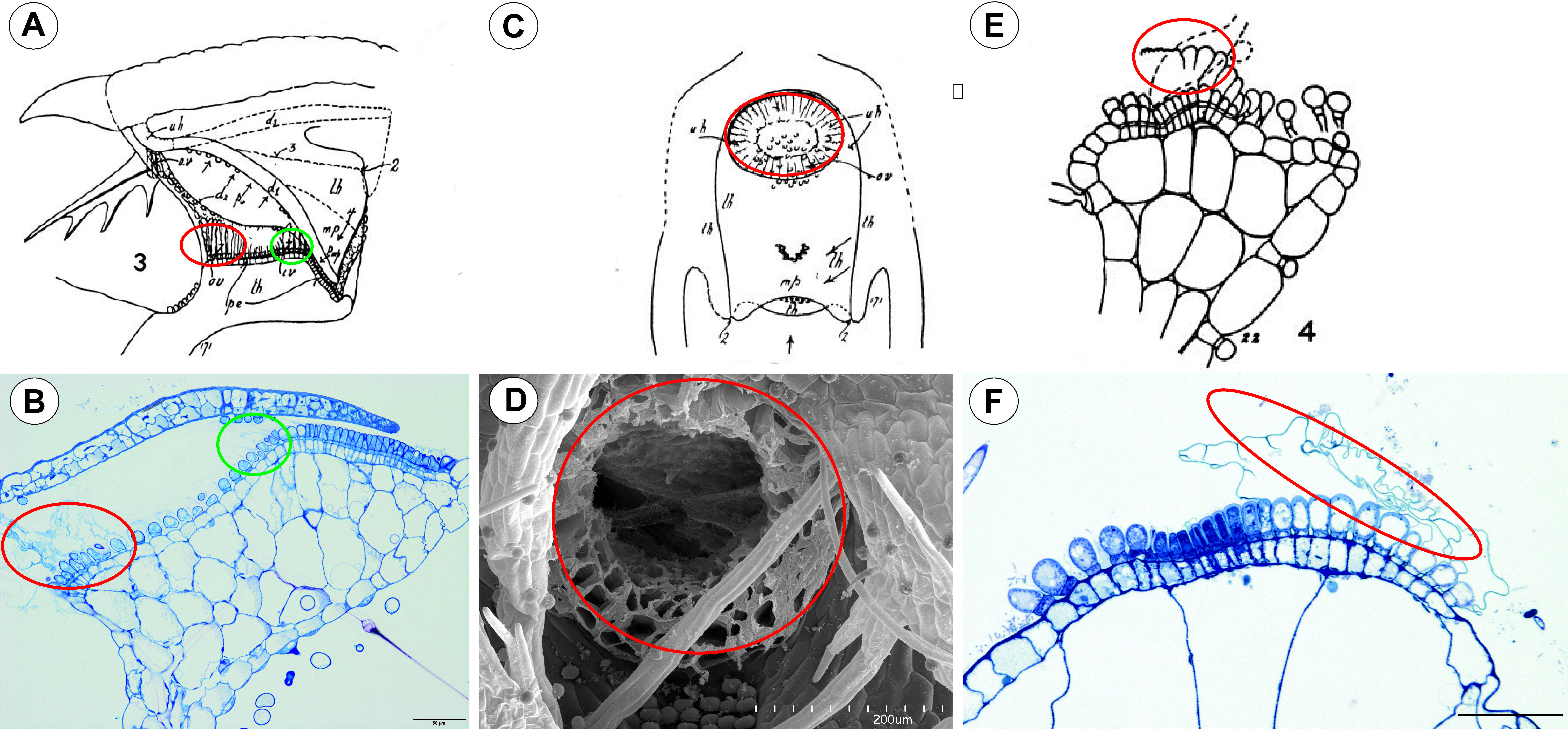

Supplement: SUPPLEMENTARY FIGURE 1 — Comparison of Lloyd’s Utricularia trap (1942) drawings with our documentation; the same structures are marked by eclipses. (A) Section of Utricularia monanthos trap entrance; the outer velum (red ellipse), the inner velum (green ellipse). (B) Utricularia dichotoma, a semi-thin longitudinal section of the threshold showing the outer velum (red ellipse), the inner velum (green ellipse); scale bar = 50 μm. (C) Utricularia monanthos, trap opening is guarded by the outer velum (red ellipse). (D) Utricularia novae-zelandiae, view of a trap entrance showing the ring of the velum (red circle); scale bar = 200 μm. (E) Transverse selection of the threshold showing velum (red ellipse) of Utricularia vulgaris trap type. (F) Utricularia reflexa, a semi-thin longitudinal section of the threshold showing velum (red ellipse); scale bar = 50 μm. [file Image_1.JPEG]
